# The Integration of Interlinkages Between Nature and Human Health in Primary Health Care: Protocol for a Scoping Review

**DOI:** 10.2196/12510

**Published:** 2019-01-18

**Authors:** Laura Lauwers, Hilde Bastiaens, Roy Remmen, Hans Keune

**Affiliations:** 1 Department of Primary and Interdisciplinary Care Faculty of Medicine and Health Sciences University of Antwerp Antwerp Belgium; 2 Belgian Biodiversity Platform Nature and Society Team Research Institute for Nature and Forest Research Brussels Belgium

**Keywords:** primary health care, nature, health, human microbiome, infectious diseases, natural disasters, medicinal plants, nutrition, nature-based care

## Abstract

**Background:**

International overview reports and the majority of scientific publications on interlinkages between nature and human health (NHI) do not seem to focus on the role of the health care sector. Primary health care (PHC) is often the first point of contact people have with the health care system and provides comprehensive, accessible, and community-based care that meets the health needs of individuals throughout their life. PHC is a vital backbone for linking knowledge and practice within the organization of health care. This scoping review aims to focus on the potential role of PHC in relation to NHI.

**Objective:**

The objective of this protocol is to present the method used to scope international overview reports and scientific publications on what is mentioned on the integration of NHI in PHC.

**Methods:**

The international overview reports have been screened for keywords relating to PHC. We developed a specific search strategy to scope scientific literature on NHI in relation to PHC. The scientific literature search ran in Web of Science (WOS) and PubMed from inception to May 2017. The scientific publications are screened by 2 independent reviewers, which will result in a list of relevant publications that meet eligibility and inclusion criteria.

**Results:**

On the basis of a first screen on the title of the first 200 results in both search engines, we decided to restrict to WOS. First insights in the international overview reports and the quantitative overview of the results in WOS give a first impression of a missing link between NHI and PHC. The findings are expected to identify knowledge gaps in the translation of evidence on NHI in PHC practices and the role of PHC regarding the application of that evidence in health care practice.

**Conclusions:**

This is, to our knowledge, the first study that seeks to relate existing knowledge on NHI to PHC. The presentation of our method through this protocol allows researchers to build upon and improve our work in future research on the practical implementation of NIH. The findings of the scoping review are expected to guide future scientific research, international policy directives, and PHC workers to fill the gaps in the integration of NHI in PHC.

**International Registered Report Identifier (IRRID):**

DERR1-10.2196/12510

## Introduction

### Background

**Figure 1:**
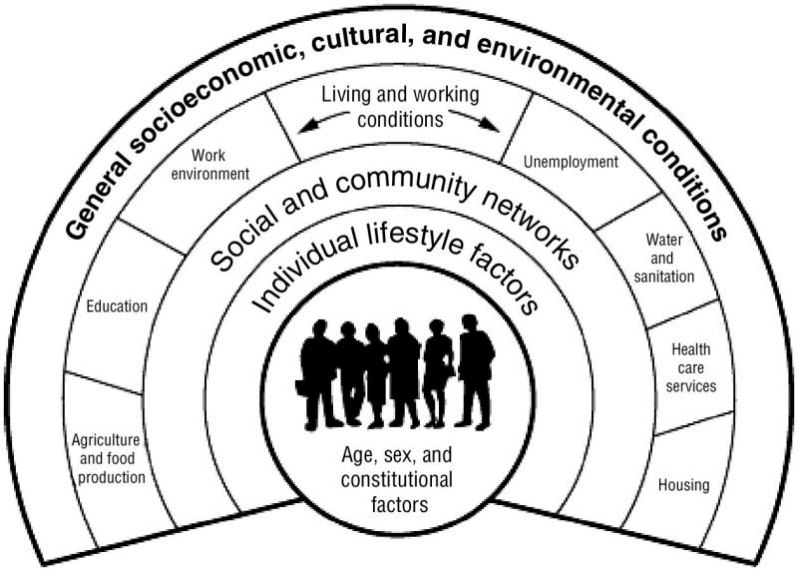
The Dahlgren-Whitehead model that revises the determinants of health.

The interlinkages between nature and human health (NHI) have been approached differently by health care over time and space. Where nature is considered a threat to health due to the cause of diseases associated with mass mortalities, nature simultaneously provides the medicinal resources to heal from diseases. This contrary view on NHI is still present, though the research field has expanded with changes in representing health being more than only related to morbidity and mortality and nature being more than a cause of diseases and a resource of medication [1]. Where the biomedical model still dominates in Western countries and gains interest in developing countries, new health approaches complement the central biomedical idea that health improvement mainly requires an understanding of biological causation by adding other determining factors [2]. The Dahlgren-Whitehead model portrays health as determined by a multilevel interaction running from individual to family to community to living and working social status to external forces of society, economy, culture, and environment [2] (Figure 1). This approach of human health reflects the increased attention for preventive health care complementing curative health care.

The generalist perspective makes the PHC setting an ideal partner for integrated approaches covering the multifaceted linkages between nature and human health. PHC is often the first point of contact people have with the health care system and provides comprehensive, accessible, and community-based care that meets the health needs of individuals throughout their life. This scoping review aims to focus on the potential role of PHC in relation to NHI.

Several determinants represented in Figure 1, for example, agriculture and food production and water and sanitation, coincide with the ecosystem approach of nature. Ecosystem services representing all the benefits and functions of natural ecosystems to people cannot be disentangled from health as people cannot remain healthy without clean air, clean water, food, and other resources provided [1]. However, these positive benefits should not ignore the remaining threat of natural ecosystems, such as spreading infectious diseases and contributing to toxicities and natural disasters that again have a major health impact. These new approaches of health and nature and the linkage between both have increased the interdisciplinary character of the research field. The number of scientific publications on NHI has vastly increased in recent years [1], but the majority does not seem to focus on the role of the health care sector. The World Health Organization (WHO) report titled “Ecosystems and Human Well-Being: Health Synthesis” did not specify the role of PHC in applying this knowledge [3].

### Objectives

The objective of this protocol is to perform a scoping review aimed at recent international overview reports and scientific publications on the use of knowledge on NHI in PHC. To scope the available knowledge, the following research question was developed: What does the literature mention on the integration of NHI in PHC? To do so, this scoping review screens the literature for arguments, practice supporting tools and methods, management approaches, and challenges and constraints characterizing the integration of NHI in PHC.

## Methods

### Strategy

#### Search Strategy

We searched in the grey literature for international overview reports. The protocol presents a list of international overview reports on NHI obtained through a snowballing search starting from the personal knowledge of the coauthor (HK) through his involvement in international networks and expert communities working on the theme of NHI. The international overview reports are complemented by a search in the scientific literature.

#### Sources of Knowledge

Table 1 gives an overview of the selection of recent international overview reports on NHI. The WHO contribution to the Millennium Ecosystem Assessment [3] is included to have a historical perspective. Moreover, 1 international overview report lead by the WHO and the Convention on Biological Diversity (CBD) is included in this review as it illustrates the commitment of international governmental organizations in collaboration with the scientific community. This report results from the joint initiative by the WHO and the CBD for a state-of-knowledge review on biodiversity and human health [4]. Furthermore, 2 international overview reports initiated by the European Commission (Directorate General Environment) on one hand [5] and WHO/Europe on the other hand [6] are included as they illustrate the new trend in the international interest, especially in industrialized and urbanized countries, for the potential human health benefits from nature. The first report gives an overview of the health and social benefits of nature and biodiversity protection [5]. The second report includes a review on the effects of urban green space in relation to human health [6]. Although these reports largely focus on human health benefits from nature, especially the WHO/Europe report incorporates an effort to also address potential human health risks (such as infectious disease, allergy, and injury) related to the greening of urban areas. To further address the human health risks from nature, another report led by the United Nations Environment Programme on a healthy environment [7] is included in this review. In this report, a number of international governmental organizations worked together, including the WHO and CBD and conventions related to environmental pollution, such as the Stockholm Convention on Persistent Organic Pollutants [7]. Finally, a report derived from the Rockefeller Foundation-Lancet Commission on planetary health is included as it is a more science-driven initiative reviewing the relation between nature and human health [8].

The selection of international overview reports is complemented by a search for scientific literature in Web of Science (WOS) and PubMed. The literature search run in WOS from inception to May 2017. We conducted a first search by combining the search strings for “nature” and “PHC” in both engines. The search in PubMed resulted in a higher number of publications (n=9074) than the search in WOS (n=471). As the screening on relevance of the title and abstract of the first 200 results in both databases indicated that the results in PubMed did not result in additional relevant papers to the results in WOS, the search was restricted to WOS. Else the number of papers to be assessed would make the work practically unfeasible with little added value for the outcomes of our analysis. Besides looking at nature in general in relation to PHC, we adapt the nature-health subthemes presented in the WHO-CBD report [4]: human microbiome, infectious diseases, natural disasters, medicinal plants, and nutrition. We developed an additional subtheme “nature-based care” and considered it as an umbrella term for health care interventions related to the environment or nature. The relevance of each subtheme is explained in the following paragraphs.

**Table 1:** Overview of selected international overview reports.

Report reference	Title of report	Additional information
WHO^a^ [3]	Ecosystems and Human Well-Being: Health Synthesis	Report of the Millennium Ecosystem Assessment
WHO-CBD^b^ [4]	Connecting global priorities: biodiversity and human health	State of knowledge review
ten Brink et al [5]	The Health and Social Benefits of Nature and Biodiversity Protection	Report for the European Commission-DG Environment
WHO [6]	Urban green spaces and health. A review of evidence	WHO Regional Office for Europe
UNEP^c^ [7]	Healthy Environment, Healthy People	Thematic report: Ministerial policy review
Whitmee et al [8]	Safeguarding human health in the Anthropocene epoch	Report of The Rockefeller Foundation-Lancet Commission on planetary health

^a^WHO: World Health Organization.

^b^CBD: Convention on Biological Diversity.

^c^UNEP: United Nations Environment Programme.

##### Nature-Based Care

Over the years, several concepts have been developed to grasp nature-health linkages, such as green care, green prescription, green exercise, and nature-based interventions. The concepts and how they are used or interpreted are not always referring to the environment or nature in the same manner. Horton promotes green care to stimulate general practitioners to raise awareness among their patients to maintain their health while respecting the environment and ecosystem, on which their health also depends [9]. Horton gives the examples of overconsumption in relation to both the environment and obesity and walking and cycling as forms of physical exercise that have minimal impact on the environment [9]. In a special issue on green care in the *International Journal of Therapeutic Communities*, green care is referred to as outdoor activities and nature in a therapeutic context [10]. Steigen defines the concept as “using animals, plants and nature in an active process to offer health-promoting activities for people” [11]. Sempik and Bragg define the concept as “utilizing plants, animals, and landscapes to create interventions to promote health and well-being” [12]. Haubenhofer interprets the concept similarly but explicitly linking it to “a person’s social, physical, mental, and even educational well-being,” linking “traditional healthcare and other sectors of human societies, like agriculture, gardening, landscape and nature conservation, animal keeping and animal husbandry” [13]. Barton et al promote the use of the term green exercise as an umbrella term relating healthy activity to the presence of nature [14]. Green prescription traditionally is defined as “a prescription for exercise” [15] but not specifically linking it to a natural environment [16,17]. Van den Berg particularly links it to the natural environment and emphasizes primary health care professionals as key actors in stimulating nature-based activities [18].

##### Human Microbiome

Quite some recent studies [19-21] show the importance of contact with nature for a healthy immune system. Declining contact with some forms of environmental microbiota may contribute to the rapidly increasing prevalence of allergies and other chronic inflammatory diseases among urban populations worldwide. This makes the relation between wild microbes and the human microbiome a promising field for health care research.

##### Nutrition

The contribution of nature to healthy nutrition is a well-established field of expertise: biodiversity contributes to food diversity for healthy human diets as well as supports pollination and soil fertility, which are essential to food production [22].

##### Medicinal Plants

The importance of nature for providing medicinal plants is also well established: biological resources historically contribute to health care, with medicinal plants being important for traditional medicine and international trade [23].

##### Infectious Diseases

Nature-related infectious diseases pose an important nature-related human health risk in quite some regions in the world: “Two-thirds of known human infectious pathogens have emerged from animals, with the majority of recently emerging pathogens originating in wildlife,” partly driven by anthropogenic disturbance and biodiversity loss [24].

##### Natural Disasters

Quite some regions, moreover, suffer from natural disasters: “more mid- and small-sized disasters are now occurring more often, while increasing urbanization and the threat of climate change place more focus on the future social, economic, environmental and public health impacts of natural disaster events” [25].

#### Search Strings

With the help of PHC professionals and the application of PubMed search builder, a search string for PHC has been developed (Table 2). We developed search strings for “nature” in general and the 6 nature-health subthemes described above based on the search strategy for the Regional Assessment for Europe and Central Asia by the Intergovernmental Platform on Biodiversity and Ecosystem Services (IPBES) [26]. This search strategy derives from the WHO and the CBD 2015 state of knowledge review [4] and how this was translated in search strings on biodiversity-health linkages and further fine-tuned (with coauthor HK) for the application by the IPBES. A selection of these search strings has been slightly adapted for the development of the search strings of the nature-health subthemes for this protocol. On the basis of the search strings for noncommunicable diseases, mental health and physical fitness in relation to practical nature-related interventions, and keywords present in the paper of Van den Berg, we developed the search string for the subtheme “nature-based care” [18].

First, the search strings for the 6 nature-health subthemes have been separately applied in WOS to get a quantitative view on the research interest for a certain nature-health subtheme. Second, the search strings for the 6 nature-health subthemes have been separately combined with the search string for PHC, using the Boolean operator *AND*. Currently, the results of the combinations of these search strings are being checked on relevance. First insights in the search results showed that many results deviate strongly from the focus of our scoping review and did not link to nature at all. Therefore, we decided to add an additional step in which the search string of each nature-health subtheme is combined with the search string for PHC and the search string developed for “nature” in general, using the Boolean operator *AND*. The initial combination of the search string for PHC and nature allowed to check if some nature-health–related themes were not captured by the 6 nature-health subthemes. The combinations of search strings are summarized below:

1. nature-health subtheme
2. nature-health subtheme AND primary care
3. nature-health subtheme AND primary care AND nature
4. nature AND primary care

**Table 2:** Overview of search strings applied in Web of Science.

Category		Search strings
Primary health care		“general pract*” OR “GP” OR “primary care” OR “primary health care” OR “primary healthcare” OR “family pract*” OR “family medicine” OR “family physician*” OR “family doctor*”
Nature		“biological diversity” OR biodivers* OR “living natural resource*” OR “living resource*” OR “natur* diversity” OR “diversity in nature” OR “*species diversity” OR “int*-speci* diversity” OR “genetic diversity” OR “diversity of gene*” OR ecosystem* OR “ecological system*” OR “ecosystem service*” OR “landscape service*” OR “environmental service*” OR “ecological service*” OR “natur* capital*” OR “nature based solution*” OR “environmental capital*” OR “green infrastructure” OR greenspace* OR “green space*” OR “blue infrastructure” OR bluespace* OR “blue space*” OR flora* OR fauna* OR wildlife OR “natural habitat*” OR “ecological habitat” OR “wildlife habitat*” OR “invasive * species” OR biogeograph* OR “bio-geograph*” OR “natur* space*” OR “natur* environment*”
**Nature-health subthemes**		
	Nature-based care	“green care” OR “green exercise” OR “green gym” OR “green leisure” OR “green recreation” OR “environmental care” OR “environmental exercise” OR “environmental gym” OR “environmental leisure” OR “environmental recreation” OR “restorative activit*” OR “restorative exercise*” OR “green therap*” OR “environmental therap*” OR “outdoor therap*” OR “green prescription” OR (rehab* AND garden* OR “nature-based rehabilitation” OR “nature based rehabilitation” OR (therap* AND garden*) OR (horticultur* AND therap*) OR “care farm*” OR (“walk” AND “talk” AND “coach*”) OR “health walk*”
	Microbiome	“gut microbiome” OR “gut microbiota” OR “gut micro-organisms” OR commensal microbiome” OR “commensal microbiota” OR “commensal micro-organisms” OR dermal microbiome” OR “dermal microbiota” OR “dermal micro-organisms” OR intestin* microbiome” OR “intestin* microbiota” OR “intestin* micro-organisms” OR internal microbiome” OR “internal microbiota” OR “internal micro-organisms” OR “hygiene hypothes*s” OR “biodiversity hypothes*s” OR “Old Friends mechanism*”
	Infectious diseases	“disease* of plant origin*” OR “disease* of wildlife origin*” OR “disease ecology” OR “disease driver*” OR “disease dynamics” OR “disease emergence” OR “disturbance-disease-*” OR “driver* of emergence” OR “emerg* infectious disease*” OR “emerg* disease*” OR “emerg* infect*” OR nidality OR nidus OR “pathogen ecology” OR enzootic* OR phytonos* OR phytonotic* OR synanthrop* OR “pathogen pollution” OR “amplification effect” OR “spill-over” OR “species barrier*” OR “vector-borne” OR “vector borne” OR “animal host*” OR “ ecologic* host*” OR “plant host*” OR “wildlife host*” OR “animal reservoir*” OR “ecologic* reservoir*” OR “plant reservoir*” OR “ wildlife reservoir*” OR zooanthropono* OR zoogen* OR zoonos* OR zoonotic* OR “carrier species” OR “ competent species” OR “host abundance” OR “host density” OR “host distribution” OR “host diversity” OR “parasite abundance” OR “parasite density” OR “parasite distribution” OR “parasite diversity” OR “pathogen abundance” OR “pathogen density” OR “pathogen distribution” OR “pathogen diversity” OR “reservoir abundance” OR “reservoir density” OR “ reservoir distribution” OR “reservoir diversity” OR “vector abundance” OR “vector density” OR “vector distribution” OR “vector diversity” OR “bacterial transmission” OR “disease transmission” OR “parasite transmission” OR “pathogen transmission” OR “viral transmission”
	Natural disasters	“biodiversity-disturbance-disease” OR “disease* of animal origin*” OR “disease* of plant origin*” OR “disease* of wildlife origin*” OR “disease ecology” OR “disease driver*” OR “disease dynamics” OR “disease emergence” OR “disturbance-disease-*” OR “driver* of emergence” OR “emerg* infectious disease*” OR “emerg* disease*” OR “emerg* infect*” OR nidality OR nidus OR “pathogen ecology” OR enzootic* OR phytonos* OR phytonotic* OR synanthrop* OR “pathogen pollution” OR “amplification effect” OR “spill-over” OR “species barrier*” OR “vector-borne” OR “vector borne” OR “animal host*” OR “ ecologic* host*” OR “plant host*” OR “wildlife host*” OR “animal reservoir*” OR “ecologic* reservoir*” OR “plant reservoir*” OR “ wildlife reservoir*” OR zooanthropono* OR zoogen* OR zoonos* OR zoonotic* OR “carrier species” OR “ competent species” OR “host abundance” OR “host density” OR “host distribution” OR “host diversity” OR “parasite abundance” OR “parasite density” OR “parasite distribution” OR “parasite diversity” OR “pathogen abundance” OR “pathogen density” OR “pathogen distribution” OR “pathogen diversity” OR “reservoir abundance” OR “reservoir density” OR “ reservoir distribution” OR “reservoir diversity” OR “vector abundance” OR “vector density” OR “vector distribution” OR “vector diversity” OR “bacterial transmission” OR “disease transmission” OR “parasite transmission” OR “pathogen transmission” OR “viral transmission”
	Medicinal resources	“biodiversity for medicine” OR “biological diversity for medicine” OR “biodiversity-based medicin*” OR “biodiversity-derived medicin*” OR bioprospecting OR “biodiversity-based prospecting” OR “biodiversity based prospecting” OR “prospecting from natur*” OR “medicine* from natur*” OR “medicine* derived from natur*” OR “medicinal plant*” OR “medicinal animal*” OR “medicinal fung*”
	Nutrition	“biodiversity for food” OR “biological diversity for food” OR agrobiodivers* OR “agro-biodivers*” OR “*managed agrobiodiversity” OR “crop diversity” OR “crop wild relative*” OR “dietary *diversity” OR “food *diversity” OR “diverse diet*” OR “foods of wildlife origin*” OR bushfood* OR “bush food*” OR “home garden*” OR “species used for food” OR “food resource*” OR “nutritional resource*” OR “local food species” OR “traditional food species” OR “traditional food*” OR “traditional crop*” OR “traditional variet*” OR “wild food harvest*”

### Selection of Relevant Scientific Literature

The titles and abstracts of the results of the search string combinations 2, 3, and 4 are checked on relevance by 2 independent reviewers (first reviewer: HK; second reviewer: HB or RR). Relevance is attributed if the publications approach the nature-health subthemes in accordance with their relevance to health, as described above, and if they pay attention to PHC in a nonsuperficial manner. Publications are included when explicitly relating the research findings on NHI to PHC, with references to PHC according to the keywords of the search string. Publications are excluded from further analysis when only mentioning PHC but not linking the NHI knowledge to PHC. For pragmatic reasons, publications are only checked on relevance when the number of results is not too large (below 100). On the basis of the titles and abstracts, the reviewers make for each publication 1 of following decisions: relevant, in doubt, or irrelevant. Each decision is supported by a short argumentation. In case the first reviewer defines a publication “relevant” or “irrelevant” and the second reviewer agrees with this decision, the publication will be either included in or excluded from the scoping review. In case the first reviewer is in doubt or the second reviewer is not convinced by the decision of the first reviewer, the first reviewer reads the full text of the publication to make a final decision in consultation with the second reviewer. Foreign language material, except for papers with an English abstract, will be excluded because of the cost and time involved in translating material. Although these limits have to be adopted for practical reasons, it is worth pointing out that potentially relevant papers can be missed. As this is only a scoping review, for pragmatic reasons, the quality of the papers will not be assessed. The scoping review is only meant to give an indication of the potential.

### Content Analysis

The international overview reports on NHI have been screened for the presence of the keywords included in the search string for PHC. A quantitative overview of the scientific literature has been done, but the selection of relevant publications needs to be finalized.

The content from the international overview reports and relevant scientific publications will be presented in a way to identify the main areas of interest and gaps. Information will be extracted to answer the following questions:

- Which arguments are given to engage with PHC to integrate nature-health linkages?
- Which practice supporting tools and methods for this integration are provided?
- Which management approaches are recommendable for this integration?
- Which challenges and constraints characterize this integration?

### Ethics

Ethical approval for this protocol and planned systematic review was not required.

## Results

### International Overview Reports

A first screen of the selected international overview reports on NHI through the search for the PHC keywords has shown that the role of PHC remains mainly underreported. Therefore, we have decided to screen for the additional keyword “health prof*” as we noticed that some reports only mention this more general category of health care professionals, while also potentially intending to include PHC professionals.

### Scientific Literature

Table 3 summarizes the quantitative results of the combinations of the search strings in WOS as described in the Methods section. The combination of the search string for PHC and nature resulted in a low number of publications (n=471), especially when compared with the number of publications only linked to nature (n=525,365) or only linked to PHC (n=206,256). Similarly, the combination of the search string for each nature-health subtheme with the search string for PHC separately or combined with the search string for nature resulted in a strongly reduced number of publications compared with the total number of publications found for each nature-health subtheme.

**Table 3:** Quantitative overview of the combinations of search strings applied in Web of Science.

Nature-health subthemes	Total	Reviews		Papers	
		PHC^a^	PHC+nature	PHC	PHC+nature
Nature-based care	1140	3	0	35	2
Human microbiome	5017	4	1	14	1
Nutrition	14,026	1	0	16	1
Medicinal plants	29,803	22	2	198	35
Infectious diseases	60,142	14	1	202	5
Natural disasters	49,256	72	0	901	1

^a^PHC: primary health care.

## Discussion

To our knowledge, this scoping review is the first of its kind to explore NHI in relation to PHC. The findings of this scoping review will provide a first state of the art of NHI in relation to PHC in international overview and scientific publications. By including subthemes such as “natural disasters” and “infectious diseases” besides the other subthemes, the review attempts to include both the risks and benefits related to nature’s impact on health. The quantitative overview of the scientific literature is a first indication of a missed potential in research and practice to link evidence on NHI to PHC. A content analysis of the selected literature will allow to draw lessons on the integration of NHI in PHC. The findings are expected to identify gaps in the integration of NHI in current medical practices and to orient recommendations toward needs for action and capacity building. The presentation of the protocol of the scoping review allows researchers to build upon and improve our work in future research on the practical implementation of NHI. Results synthesized and limitations to our search strategy will be disseminated by means of a published work in a peer-reviewed journal.

## Abbreviations

CBD: Convention on Biological Diversity
IPBES: Intergovernmental Platform on Biodiversity and Ecosystem Services
NHI: interlinkages between nature and human health
PHC: primary health care
UNEP: United Nations Environment Programme
WHO: World Health Organization
WOS: Web of Science
