# Correction: The Integration of Interlinkages Between Nature and Human Health in Primary Health Care: Protocol for a Scoping Review

**DOI:** 10.2196/13660

**Published:** 2019-03-07

**Authors:** Laura Lauwers, Hilde Bastiaens, Roy Remmen, Hans Keune

**Affiliations:** 1 Department of Primary and Interdisciplinary Care Faculty of Medicine and Health Sciences University of Antwerp Antwerp Belgium; 2 Belgian Biodiversity Platform Nature and Society Team Research Institute for Nature and Forest Research Brussels Belgium

During typesetting and proofreading for “The Integration of Interlinkages Between Nature and Human Health in Primary Health Care: Protocol for a Scoping Review” (JMIR Res Protoc 2019;8(1):e12510), the following sentence was erroneously deleted from the Acknowledgments section:

This study results from the project “Green light” (*in Dutch: Licht op Groen*), hosted by the University of Antwerp and funded by the Province of Antwerp in Belgium.

The aforementioned sentence has now been reinstated.

The correction will appear in the online version of the paper on the JMIR website on March 7, 2019, together with the publication of this correction notice. Because this was made after submission to PubMed, PubMed Central, and other full-text repositories, the corrected article also has been resubmitted to those repositories.

